# A Retrospective Analysis of Concomitant Spinal Injuries in the Setting of Polytrauma at a Tertiary Hospital in Singapore

**DOI:** 10.7759/cureus.108746

**Published:** 2026-05-12

**Authors:** Theophilus Hong Qiu, Cassie Yang, Shermane Yun Wei Lim, Brian Yuan-Lang Chan, Eugene Yang, Boon Chuan Pang, Woan Wui Lim, Zhi Xu Ng

**Affiliations:** 1 Orthopedics, SingHealth, Singapore, SGP; 2 Orthopedic Surgery, Singapore General Hospital, Singapore, SGP; 3 Emergency Department, National University Hospital, Singapore, SGP; 4 Neurosurgery, Khoo Teck Puat Hospital, Singapore, SGP

**Keywords:** emergency, polytrauma, spinal injury, spinal trauma, spine, trauma

## Abstract

Aims: Polytrauma involving multiple organ systems is a pertinent health concern. Spinal injuries are often observed in polytrauma, with serious consequences to patients’ quality of life and a significant healthcare burden. Due to its complexity, there are limited studies analyzing the population of spinal injuries in polytrauma in Singapore. This study aims to retrospectively evaluate a cohort of polytrauma patients and characterize the relationship between the presence of traumatic spinal injury and clinical outcomes.

Methods: A retrospective cohort of patients who presented to the emergency department for multisystem trauma was assembled from a local trauma database at a tertiary hospital in Singapore from January 2012 to June 2020. Variables analyzed were the presence of concomitant spine injury, mechanism of injury, injury severity score (ISS), gender, age, ethnicity, and year of admission. Outcomes were mortality, length of stay, and cost.

Results: 1568 patients involved in multisystem trauma were identified for this study. It was observed that 27.2% (n=427) had concomitant spinal injuries, which were most commonly due to falls (83.6%, n=357), followed by vehicular accidents (12.9%, n=55). The percentage of falls as the primary injury mechanism increased from 25.9% in the 21- to 30-year-old age range to 96.4% in the 81- to 90-year-old age range. Meanwhile, the percentage of vehicular accidents as the primary injury mechanism decreased from 67.8% in the 21- to 30-year-old age range to 3.2% in the 81- to 90-year-old age range. Notably, the mortality rate of patients with spine injuries (2.6%, n=416, relative risk 0.17) was significantly lower than that of those without spine involvement (15.1%, n=969, p<0.001), with an adjusted odds ratio for mortality of 0.25 (95% CI (0.11, 0.52), p<0.001). The mean total length of stay was lower in patients with spinal injuries, at 11.8 days (SD 14.6, p<0.001), compared to 16.0 days (SD 28.0) in patients without spinal injuries. The mean total hospital bill was lower in patients with spinal injuries, at 8692 Singapore dollars (SD 13980, p<0.001), compared to 16359 Singapore dollars (SD 30658) in patients without spinal injuries.

Conclusions: Spinal injury in the context of polytrauma is not an independent predictor of poorer outcomes, as it has been observed to have lower mortality compared to polytrauma patients without spinal involvement. This may be attributed to the complex nature of polytrauma; previous studies have found that high-energy traumatic injuries tend to be associated with more independent living and mobility than low-energy injuries, with the former having better functional outcomes postinjury. Similarly, patients with spinal cord involvement in polytrauma, which suggests a high-energy injury, may have greater functional demands and subsequently better reserves, thus conferring better survivability.

## Introduction

Traumatic injuries are a major cause of mortality worldwide. Globally, 8% of deaths are caused by injuries, with a crude death rate of 57.2 per 100,000 population [1]. In Singapore, the Ministry of Health lists "External Causes of Morbidity and Mortality" as the fifth principal cause of death in Singapore [2].

Due to Singapore’s small land mass, trauma patients are always within a 1-hour radius of the nearest trauma center. This ensures that patients receive adequate trauma care within the “Golden Hour” for optimal outcomes [3]. There are eight public hospitals geographically spread across the island, and each institution’s Accident and Emergency (A&E) department is fully equipped to provide resuscitation, stabilization, and treatment for adult and pediatric trauma [4]. For transfers, the Singapore Civil Defence Force (SCDF) conveys patients requiring emergency care to the nearest public hospital within travel time, as designated by the Ministry of Health [5].

Spinal injury in trauma is a concerning phenomenon due to its incidence and mortality. The global burden of such injuries is estimated at 10.5 per 100,000 persons annually [6], with reported mortality rates from spinal injuries ranging from 4.4% to 16.7% [7]. They tend to be associated with high-energy trauma, often involving multiple body systems. A systematic review found that the most common causes of traumatic spinal injuries are motor vehicle collisions, followed by falls [8].

The aim of this study is to analyze the composition of concomitant spinal injuries within the local population and to characterize their significance on clinical outcomes in the setting of polytrauma. This will aid in better decision-making and planning in the management of patients with polytrauma.

There is little information on the outcomes of such patients in the local setting. More information on outcomes of spinal trauma will allow emergency physicians and trauma specialists to better triage and counsel trauma patients. For institutions, this is crucial for optimizing clinical pathways and resource allocation.

This study was previously presented as an abstract at the Global Spine Congress 2023 [9].

## Materials and methods

Study design

A retrospective cohort was assembled from a database of all patients who presented to the emergency department of a tertiary hospital in Singapore from January 2012 to June 2020. Institutional Review Board exemption was approved by the National Healthcare Group Domain Specific Review Board (approval 2022/00940).

Data collection

Collection of data from patients was performed with informed consent, in line with the Seventh Revision of the World Medical Association Declaration of Helsinki. During this period, the patients’ case details were obtained and anonymized by an appointed registered trauma nurse and keyed into an electronic spreadsheet (Microsoft, Inc., Redmond, Washington) to obtain a comprehensive trauma database.

Using Cochran’s formula with a 95% confidence interval, a minimum sample size of 384 would be required for adequate representation of the study population [10].

Patient recruitment

This study included patients from the trauma database who presented with multisystem traumatic injuries from any mechanism and fulfilled an injury severity score (ISS) of at least 9 (moderate injury and above) [11], regardless of eventual mortality outcomes. To ensure that the population obtained was focused on analyzing spinal injuries in polytrauma, the following exclusion criteria were applied: patients who had an ISS below 9, were transferred from other institutions, or had an injury involving a single body region, such as an isolated vertebral body compression fracture. Each injury sustained was coded based on the abbreviated injury score (AIS) code and then categorized into subcategories of “Vehicular Accident,” “Fall,” and “Others.” ISS was calculated as the sum of the squares of the highest AIS score (1-5) in each of the three most affected body regions. ISS must be between 1 (minimum) and 75 (maximum).

Patients were classified as having spinal injuries if their final diagnosis text included mention of injury to the vertebrae, any section of the spine (cervical, thoracic, lumbar, sacral), spinal ligaments, and spinal cord.

Statistical analysis

Analysis was performed with R version 4.1.2 (R Foundation, Vienna, Austria). The primary predictor variable was the presence of concomitant spine trauma. Other predictor variables included mechanism of injury, ISS, gender, age, ethnicity, and year of admission.

In the unadjusted comparison analyses of predictor and outcome variables in patients with and without spine injury, chi-squared tests were used to compare categorical variables, and independent t-tests were used to compare continuous variables. P-values of <0.05 were considered statistically significant. Multivariate logistic regression was then performed with mortality as the outcome to compare the adjusted odds ratios in patients with and without spine trauma. Multivariate logistic regression was performed for length of stay and cost of stay as outcomes; however, these were not included in our results, as there were no significant (p<0.05) adjusted odds ratios for patients with and without spine trauma.

Outcome variables included in this analysis were mortality, length of stay, and overall cost of admission.

## Results

A total of 1573 patients met the inclusion criteria. Of these, 1568 patients were included in the data analysis, and five patients were excluded due to incomplete data.

This cohort was made up of 58.8% (n=922) male and 41.2% (n=646) female patients (Table 1).

**Table 1:** Cohort demographics Demographic representation of patients (n=1568) presenting to the Khoo Teck Phuat Hospital Emergency Department with polytrauma, including spinal injuries. ISS, injury severity score

	Whole sample (N=1568)		Spine injury (N=427)		No spine injury (N=1141)	
	N	%	N	%	N	%
Gender						
Male	922	58.8	202	47.3	720	63.1
Female	646	41.2	225	52.7	421	36.9
Age						
≤20	53	3.4	10	2.3	43	3.8
21 to 30	255	16.3	36	8.4	219	19.2
31 to 40	162	10.3	20	4.7	142	12.4
41 to 50	153	9.8	33	7.7	120	10.5
51 to 60	204	13.0	53	12.4	151	13.2
61 to 70	206	13.1	71	16.6	135	11.8
71 to 80	244	15.6	93	21.8	151	13.2
81 to 90	252	16.1	94	22.0	158	13.8
>90	39	2.5	17	4.0	22	1.9
Ethnicity						
Chinese	1048	66.8	298	69.8	750	65.7
Malay	275	17.5	63	14.8	212	18.6
Indian	198	12.6	52	12.2	146	12.8
Others	47	3.0	14	3.3	33	2.9
Year of admission						
2012	151	9.6	53	12.4	98	8.6
2013	174	11.1	52	12.2	122	10.7
2014	194	12.4	56	13.1	138	12.1
2015	207	13.2	56	13.1	151	13.2
2016	192	12.2	52	12.2	140	12.3
2017	211	13.5	57	13.3	154	13.5
2018	190	12.1	53	12.4	137	12.0
2019	196	12.5	34	8.0	162	14.2
2020	53	3.4	14	3.3	39	3.4
Mechanism of injury						
Vehicular accidents	560	35.7	55	12.9	505	48.3
Falls	938	59.8	357	83.6	581	50.9
Others	70	4.5	15	3.5	55	4.8
ISS						
9 to 15	749	47.8	295	69.1	454	39.8
16 to 24	460	29.3	103	24.1	357	31.3
≥25	359	22.9	29	6.8	330	28.9

**Figure 1:**
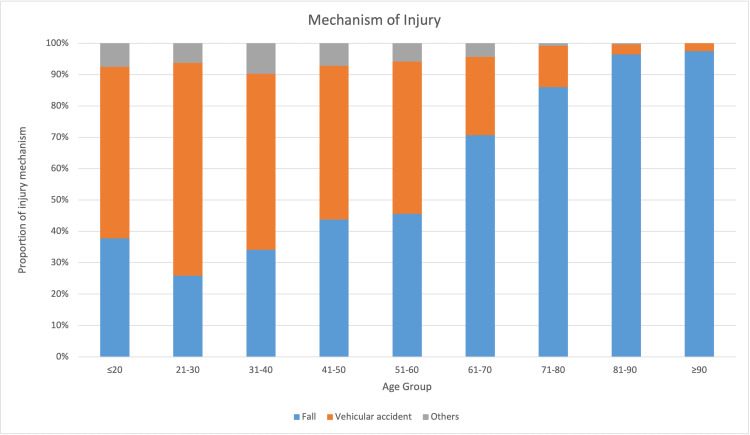
Mechanism of injury Mechanism of Injury resulting in polytrauma. Younger patients were more likely to present with polytrauma from road traffic accidents, while older patients were more likely to present with polytrauma after falls.

Most injuries were due to falls (59.8%, n=938), followed by vehicular accidents (35.7%, n=560). Trauma due to other injury mechanisms, such as interpersonal violence, machinery incidents, and others, made up a comparatively small proportion (4.46%, n=70) of patients with polytrauma. There were two peak age groups in the analyzed cohort. 16.3% of the cohort were between 21 and 30 years old, while 16.1% fell within the 81- to 90-year-old age range. With regard to the mechanism of injury within each individual age group, the percentage of falls increased from 25.9% in the 21- to 30-year-old age range to 96.4% in the 81- to 90-year-old age range. Meanwhile, the percentage of vehicular accidents decreased from 67.8% in the 21- to 30-year-old age range to 3.2% in the 81- to 90-year-old age range (Figure 1).

Of the 1568 patients included, 27.2% (n=427) had concomitant spinal injuries. A significant proportion of concomitant spinal injuries were due to falls (83.6%, n=357), followed by vehicular accidents (12.9%, n=55). In the group of patients with concomitant spinal injuries, the majority (52.7%, n=225) were female. Among patients with concomitant spinal injuries, the older age groups of 81- to 90-year-olds (22.0%, n=94), 71- to 80-year-olds (21.8%, n=93), and 61- to 70-year-olds (16.6%, n=71) made up the greatest numbers. Patients aged 20 years and below made up the smallest number of patients in this group (2.3%, n=10).

ISS was lower in patients with concomitant spinal injuries (6.8% vs. 28.9% ISS ≥25, p<0.001).

Comparatively, patients without concomitant spinal injuries were more likely to be male (63.1%, n=720) and younger. Most patients presenting without concomitant spinal injuries were in the 21- to 30-year-old age range (19.2%, n=219). The incidence of falls (50.9%, n=581) and vehicular accidents (44.3%, n=505) was also fairly equal.

Patients with concomitant spinal injuries were less likely (2.6%, n=416) to pass away during the index admission compared to patients with non-spine involvement (15.1%, n=969), with a relative risk of 0.17 (Table 2). Total length of stay was lower in patients with concomitant spinal injuries at 11.8 (14.6) days (mean, SD) compared to patients with no spine injury at 16.0 (28.0) days. Correspondingly, the total cost of stay was lower in patients with concomitant spinal injuries at 8692 (13980) Singapore dollars (mean, SD) compared to 16359 (30658) in patients without concomitant spinal injuries.

**Table 2:** Outcomes of patients presenting with and without spinal injury Mortality rate was lower in patients with spinal injury. Total length of stay and, consequently, cost of stay were lower in patients with spinal injury.

	Whole sample (N=1568)	Spine injury (N=427)	No spine injury (N=1141)	p-value
Mortality				<0.001
Alive	1385	416	969	Not applicable
Deceased	183	11	172	Not applicable
Total length of stay (days, significant difference)	14.8, 25.2	11.8, 14.6	16.0, 28.0	<0.001
Medical cost (Singapore dollars, significant difference)	14271, 27360	8692, 13980	16359, 30658	<0.001

After multivariate logistic regression was performed, concomitant spinal injuries demonstrated a significant negative association (OR=0.25, 95% CI (0.11, 0.52)) with mortality outcome (Table 3). Patients in the 71- to 80-year-old (OR=3.29, 95% CI (1.10, 10.29)) and 81- to 90-year-old (OR=5.99, 95% CI (1.81, 20.49)) age groups had significantly elevated odds of mortality compared to the reference age group of under 20 years old. Other factors that had a significant association with mortality were ISS. ISS scores of 16-24 (“severe”) had an OR=2.86 (95% CI (1.16, 7.31)), and ISS scores of 25 or more (“profound”) had an OR=188.67 (95% CI (82.60, 487.36)).

**Table 3:** Odds ratio for mortality grouped by gender, age, ethnicity, admission year, mechanism of injury, ISS, and spinal injury Concomitant spinal injuries in polytrauma patients demonstrated a significant negative association (OR=0.25, 95% CI [0.11, 0.52]) with mortality outcome. ISS, injury severity score

	Odds ratio	95% confidence interval	p-value
Gender			
Female	Reference		
Male	1.36	(0.85, 2.18)	0.204
Age			
≤20	Reference		
21 to 30	0.84	(0.32, 2.26)	0.719
31 to 40	0.80	(0.29, 2.31)	0.674
41 to 50	1.13	(0.39, 3.38)	0.827
51 to 60	1.73	(0.63, 4.93)	0.293
61 to 70	1.57	(0.55, 4.69)	0.405
71 to 80	3.29	(1.10, 10.29)	0.036*
81 to 90	5.99	(1.81, 20.49)	0.004**
>90	1.63	(0.16, 12.60)	0.658
Ethnicity			
Chinese	Reference		
Malay	0.93	(0.53, 1.61)	0.801
Indian	1.51	(1.22, 2.76)	0.185
Others	0.22	(0.04, 0.82)	0.037*
Year of admission			
2012	Reference		
2013	1.13	(0.50, 2.55)	0.765
2014	0.57	(0.23, 1.38)	0.219
2015	0.99	(0.44, 2.24)	0.981
2016	0.75	(0.32, 1.74)	0.499
2017	0.54	(0.24, 1.20)	0.131
2018	0.70	(0.31, 1.57)	0.389
2019	0.50	(0.22, 1.09)	0.082
2020	0.95	(0.27, 3.20)	0.936
Mechanism of injury			
Vehicular accidents	Reference		
Falls	2.48	(1.54. 4.04)	<0.001***
Others	0.50	(0.17, 1.33)	0.187
ISS			
9 to 15	Reference		
16 to 24	2.86	(1.16, 7.31)	0.023*
≥25	188.67	(82.60. 487.36)	<0.001***
Traumatic spinal injury	0.25	(0.11, 0.52)	<0.001***

## Discussion

The notable finding is that, in the context of local polytrauma cases, concomitant spinal injury is not an independent predictor of increased mortality. It was noted that patients with concomitant spinal injuries did not have higher mortality during the index admission than patients without concomitant spinal injuries. Conversely, in this study cohort, concomitant spinal injury was an independent predictor of lower mortality in patients with polytrauma.

This finding may seem counterintuitive, given that concomitant spine injuries often occur in the setting of high-energy trauma, which would suggest poorer outcomes. However, there have been similar observations regarding the association between concomitant spinal injuries in polytrauma and mortality. Tee et al. [7] reported that although unadjusted spine injuries suggested a trend toward increased mortality, after adjustment for confounders of mortality, the presence of spinal injuries was not shown to predict mortality. Galganski et al. [12] also showed that in burn trauma victims, spinal cord injury did not affect mortality outcomes, whereas the mechanism and severity of injuries had a more significant impact on mortality. Riemann et al. [13] also found that the combination of a traumatic spinal injury with other systemic injuries was not a significant predictor of death. This is also reflected in the results of this study, which demonstrated that the mechanism of injury and higher ISS were better predictors of mortality.

The presence of certain factors that allowed patients to survive the initial trauma may also positively influence survival outcomes. Such factors include age and active lifestyle, which thereby confer better baseline function and greater reserves. Shah et al. demonstrated that geriatric patients who sustained high-energy traumatic injury had better functional outcomes than those with low-energy traumatic injury; this was attributed to the complex nature of sustaining a high-energy injury, and it was found that patients with high-energy trauma were more likely to be living independently and had better preinjury mobility compared to patients with low-energy trauma [14]. This finding may be applied to this study, which could suggest that patients who survive the index admission with polytrauma and spinal involvement have better functional reserves, thus conferring better survival outcomes.

Another pertinent finding is that the main mechanism of traumatic injuries differs between age groups. In this study, it was found that there was an increased incidence of falls among the elderly compared to younger populations (25.9% in the 21- to 30-year-old age range and 96.4% in the 81- to 90-year-old age range). On the other hand, there was a greater incidence of vehicular accidents among younger populations than the elderly (67.8% in the 21- to 30-year-old age range and 3.2% in the 81- to 90-year-old age range). While there has been no recent epidemiological study of trauma patients in Singapore, the authors noted a 2014 study from the same institution that described falls as the more common cause of traumatic injury in the elderly population, while vehicular accidents were the main cause of trauma in the younger population [15]. This suggests that preinjury lifestyle and functional reserves may predispose patients to a particular mechanism of injury, and further studies to characterize this may be useful in suggesting precautionary measures to reduce the incidence of such injuries in different age groups.

The demographics of this study cohort differ slightly from those of existing studies. Existing literature from other populations suggests that males make up a greater proportion of patients presenting with spine injury in trauma settings [16], whereas this cohort had more females in this category. Furthermore, patients presenting with spine injury in the setting of polytrauma in this study were generally older than those seen in other epidemiological studies of trauma [7,17]. The authors note that these study populations did not only include spinal trauma patients with polytrauma; hence, any comparisons made are nonempirical in nature.

Limitations

One important confounder is that prehospital mortality was not included in this study. Patients who have multisystem injuries and spinal injuries may be pronounced dead at the scene and are thus inadvertently excluded from the study cohort. This would have led to selection bias and hindered the objective analysis of the outcomes of spinal injury in polytrauma.

Our cohort of patients with spine trauma was well represented in “moderate” and “severe” injuries based on ISS scores. However, a confounder would be that patients with “profound” injuries based on an ISS score of ≥25 only accounted for 6.8% of patients with spinal trauma. Patients in this group who died may have had undiagnosed spinal trauma if they could not be imaged (x-ray, computed tomography, magnetic resonance imaging) prior to their death.

The study did not analyze the specific management of trauma patients after admission. As there is no fixed protocol for management of spinal injuries in the setting of multisystem injury in the study hospital, patients may have been managed by different specialties with different approaches. This could lead to varying outcomes. Furthermore, there were no data with regard to postadmission complications, such as acute myocardial infarction, adult respiratory distress syndrome, and deep vein thrombosis, which are known to have significant mortality risks [18]. The development and subsequent management of these complications may have contributed to mortality in ways independent of the initial mechanism of injury; thus, their exclusion may limit the accuracy of the study’s correlation between mechanism of injury, type of injury sustained, and subsequent mortality.

The authors suggest that future research is required to verify the findings reported in this study. Including trauma victims who do not survive to be admitted to the hospital would allow a more objective analysis of the survivability of spinal injury in polytrauma. Another area of potential future research is the implementation of preventive strategies for age-range-specific mechanisms of injury, as this study has shown a pattern in the type of injuries sustained across different age groups.

## Conclusions

This single retrospective analysis of an eight-year dataset suggests that spinal involvement in polytrauma for patients treated at a tertiary institution does not result in poorer patient outcomes. Instead, spinal involvement has a negative association with mortality. As discussed above, these results would benefit from data on both prehospital mortality and the ability to adequately image and diagnose all patients presenting to the emergency department. Confirmation through comparison with outcomes in other institutions and studies will be beneficial.

The findings above would be best applied to polytrauma patients presenting to the emergency department with multisystem injuries, including diagnosed spine trauma. These patients, once stabilized, are likely to be evaluated for safe definitive surgery. The choice and timing of surgical interventions should be tailored to each patient’s injury status and avoid increasing patient morbidity. A better understanding of spine trauma in these patients and how it affects patient outcomes would provide the medical team with more information when prioritizing interventions. This study also finds that falls are the more common cause of traumatic injury in the elderly, while vehicular accidents are more common in younger patients, and future studies are encouraged to suggest age-specific preventive measures.
